# Peutz-Jeghers syndrome in women with jejunojejunal intussusception and multiple gastrointestinal polyposis: A case report

**DOI:** 10.1016/j.ijscr.2024.110713

**Published:** 2024-12-01

**Authors:** Samrat Shrestha, Bijay Raj Bhatta, Mecklina Shrestha, Kaushal S. Thapa

**Affiliations:** a National Academy of Medical Sciences, NAMS, Bir Hospital, Department of General Surgery, Kathmandu, Nepal; b College of Medical Sciences (CoMS), Department of Emergency Medicine, Bharatpur, Nepal

**Keywords:** Peutz-Jeghers syndrome, Intussusception, Polyp, Laparotomy, Case report

## Abstract

**Introduction and importance:**

Peutz-Jeghers syndrome (PJS) is a rare autosomal dominant disorder characterized by multiple gastrointestinal hamartomatous polyposis, mucocutaneous pigmentation, and/or a family history of PJS. Intussusception in patient with PJS is a common complication presenting as abdominal pain and a feature of intestinal obstruction secondary to polyps.

**Case presentation:**

A 36-year-old woman presented to the emergency department with bilateral flank pain, melena, and generalized fatigue with multiple, black-pigmented lesions on her lips extending to mucosa of buccal cavity. Contrast-enhanced computed tomography confirmed multiple gastrointestinal polyps with jejuno-jejunal intussusception. Exploratory laparotomy revealed jejuno-jejunal intussusception with multiple pedunculated polyps. Segmental jejunal resection followed by jejunojejunal stapled anastomosis was performed. Histopathological examination revealed a hamartomatous polyp, confirming the diagnosis of PJS.

**Clinical discussion:**

PJS is a rare autosomal disorder due to a mutation in the tumor suppressor gene STK11, found on chromosome 19p13. The estimated prevalence of PJS ranges between 1 in 8300 and 1 in 280,000. Intussusception is one of the most common complications, occurring in almost half the patients. Surgical resection remains the recommended treatment for rapidly growing polyps associated with intussusception. Screening at regular intervals for early detection of cancers and recurrence of polyps after excision is recommended.

**Conclusion:**

Accurate diagnosis of PJS depends on childhood history, family history, physical examination, endoscopic evaluation, and genetic testing. Their presentation varies, ranging from gastointestinal bleeding to intestinal obstruction brought on by intussusception. Surgical resections remain the recommended treatment in patients with intussusception associated with large and rapidly growing polyps.

## Highlights

• Intussusception is rare in adults; mostly tumor-related; Peutz-Jeghers syndrome is one of the rare causes.
• Hamartomatous polyposis, mucocutaneous pigmentation, and/or positive family history are pathognomonic of PJS.
• There is a risk of intestinal obstruction, and intestinal or extraintestinal malignancies in patients with PJS.
• Surgical resection is recommended, with a “clean sweep” approach.

## 1 Introduction

Intussusception is one of the causes of intestinal obstruction in an adult with no gender disparity. Enteroenteric is the most common type (45.5 %), followed by ileocolic (34.1 %), colocolic (18.2 %), and sigmoidorectal (2.3 %) [1]. Among them, approximately 63 % of the cases are tumor-related [1]. Peutz-Jeghers syndrome (PJS) with hamartomatous polyp is one of the rare causes of intussusception, affecting 1 in every 100,000 people [2]. Apart from polyps, this genetic disorder is characterized by distinctive melanotic macules and a heightened risk of multiple cancers. Due to the potential for several complications like bleeding, intussusception and intestinal obstruction, early diagnosis, and regular surveillance are critical for managing this condition [2]. We are reporting a similar case of a 36-year-old female with recurrent colicky abdominal pain, vomiting and melena for a year with mucocutaneous pigmentations of lips and buccal mucosa who presented with feature of intestinal obstruction. Clinical diagnosis of PJS with jejuno-jejunal intussusception was confirmed on imaging studies and by histopathological analysis. This case report has been reported according to the revised SCARE guidelines, 2023 [3].

## 2 Case presentation

**Fig. 1:**
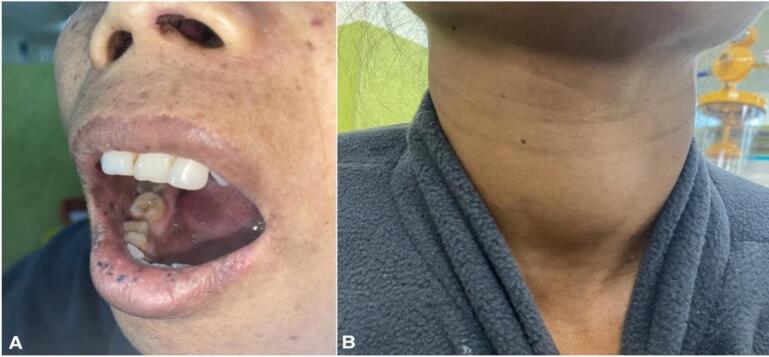
A: Dark mucocutaneous pigmented macules over the lower lip and buccal mucosa. B. Thyroid swelling.

A 36-year-old woman arrived at the emergency department complaining of abdominal pain over her bilateral flank, accompanied by black stool and a per rectal bleed that had been ongoing for the past year. The patient also had multiple episodes of vomiting with generalized fatigue and weakness. Additionally, the patient had multiple black pigmented lesions over the lower lip (Fig. 1A) since childhood and midline neck swelling (Fig. 1B) for 2 years. She had a history of lung cancer in her brother who died at the age of 45 years, rest family history was not significant. On physical examination, the patient was tachycardic with a pulse rate of 116 beats per minute; the rest of the vitals were within normal limits. The patient was pale; abdomen was distended and tenderness over the periumbilical and lumbar regions of the abdomen on palpation. Upon systemic examination, dark mucocutaneous pigmented macules over the perioral region extending to buccal mucosa, along with a thyroid nodule of 5*5 cm and a left breast lump, were noted. Laboratory parameters revealed anemia (hemoglobin level 6 g/dl). The patient had multiple blood transfusions over the last year; the recent transfusion was 2 months ago, and the rest of the parameters were within the normal limit. Blood transfusions and other supportive management were done to stabilize the patient.

**Fig. 2:**
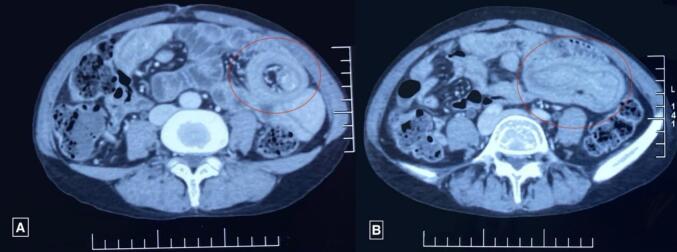
CECT Abdomen-Axial Section showing target sign (A: red circle) and jejunojejunal intussusception (B: red circle). CECT: Contrast-Enhanced Computed Tomography.

**Fig. 3:**
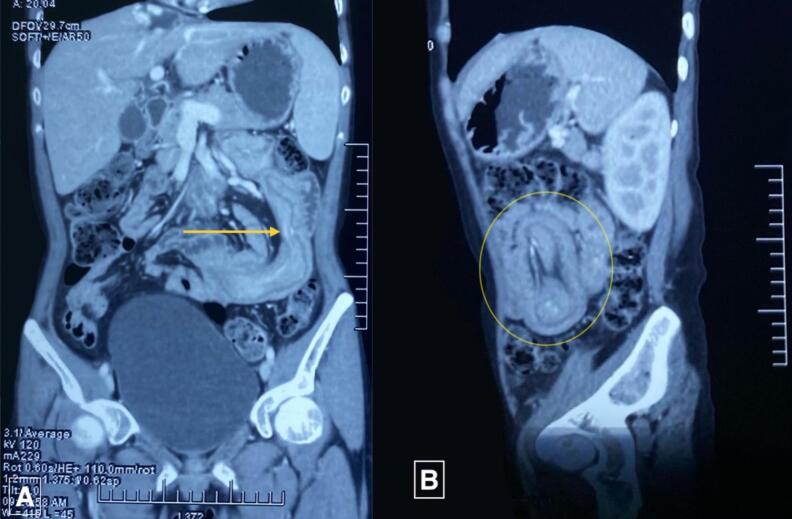
CECT Abdomen showing sites of intussusception in coronal and sagittal sections (yellow arrow in panel A and yellow circle in panel B). CECT: Contrast-Enhanced Computed Tomography.

**Fig. 4:**
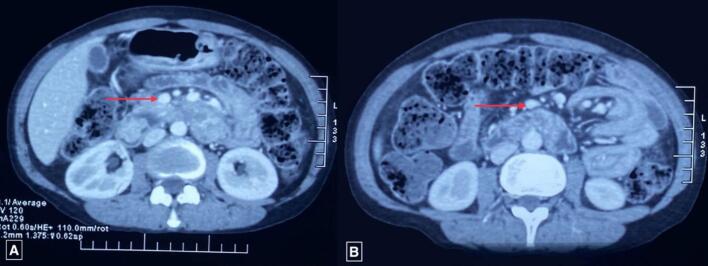
CECT Abdomen-Axial Section showing enhancing soft tissue polypoidal lesion at duodenum (A: red arrow) and at jejunum (B: red arrow). CECT: Contrast-Enhanced Computed Tomography.

Ultrasonography (USG) of the abdomen revealed a target sign with telescoping of the proximal jejunal segment over the distal segment giving a pseudo-kidney sign, with a length span of >6 cm suggesting jejuno-jejunal intussusception. Contrast-enhanced computed tomography (CECT) revealed multiple polypoidal masses in the 2nd, 3rd, and 4th parts of the duodenum, the largest measuring 23*21 mm, and 3 jejunal polyps, the largest measuring 41*22 mm (Fig. 2), with jejuno-jejunal intussusception at 3 sites (Fig. 3, Fig. 4). Multiple hemangiomas were noted in the liver. Upper gastrointestinal(UGI) endoscopy revealed gastroduodenal polyps, and colonoscopy showed 3 20*20 mm pedunculated polyps in the rectum. USG neck showed an oval spongiform nodule with calcification (TIRADS-2) and mammography showed multiple equal-density lesions in the upper outer quadrant of the left breast, which on USG correlation showed multiple cystic lesions (BIRADS-2). A provisional diagnosis of PJS was made.

**Fig. 5:**
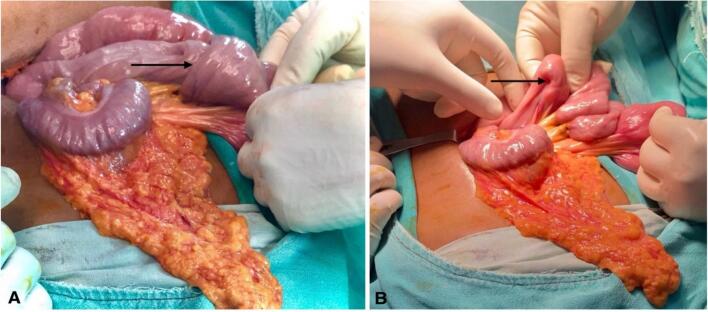
A: Jejuno-jejunal intussusception (black arrow) B: Serosal indentation (black arrow) due to polyp serving as a lead point.

**Fig. 6:**
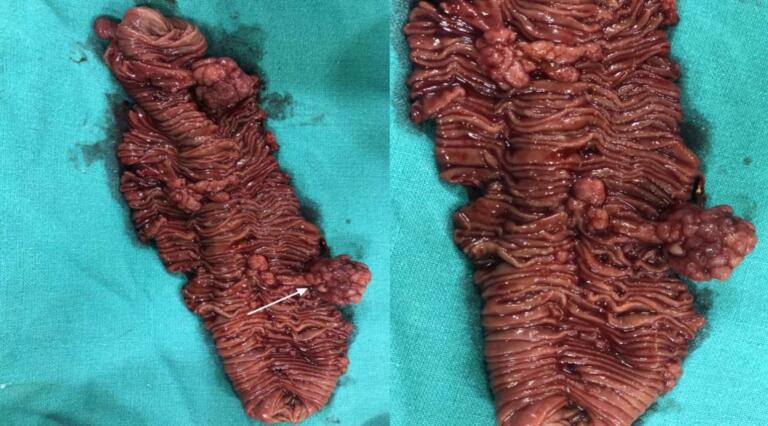
Resected specimen of jejunum showing multiple pedunculated polyps (white arrow) in its lumen.

Since the patient had unrelieved abdominal pain with vomiting and persistent intussusception on review USG, an exploratory laparotomy was scheduled. Intraoperatively, jejuno-jejunal intussusception (15 cm distal to the ligament of Treitz) (Fig. 5A) with pedunculated polyps served as a lead point (Fig. 5B), which was revealed on enterotomy. There were multiple polyps (3 larger polyps and multiple smaller polyps) in the jejunum spanning about 20 cm of the jejunum (Fig. 6), so segmental jejunal resection was done, followed by side-to-side jejuno-jejunal stapled anastomosis.

**Fig. 7:**
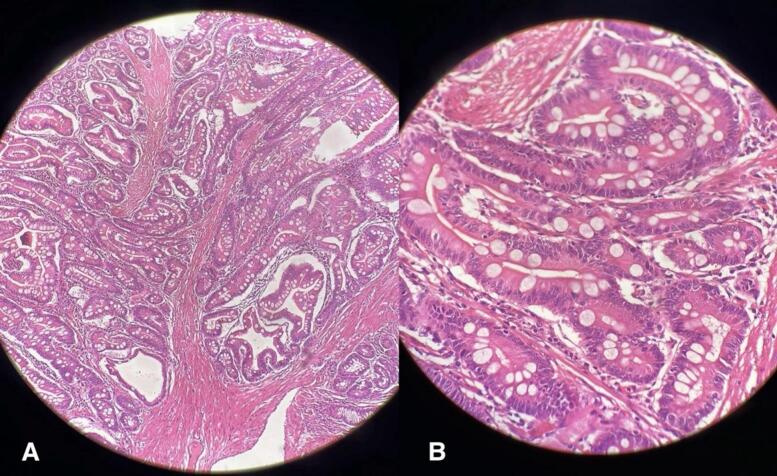
Histopathological examination showing papillary villous architecture with tree-like arborization of compact smooth muscle bundle surrounded by goblet cells. A: 40× magnification; B: 100× magnification.

Histopathological examination of the specimen revealed a hamartomatous polyp without a feature of dysplasia (Fig. 7), characteristic of PJS. The patient's postoperative course was uneventful and was discharged on the 5th postoperative day and advised for regular follow-up.

The patient is on regular follow-up in the outpatient department without any symptoms and radiologically confirmed no feature of intussusception or intestinal obstruction. The rectal polyp was removed on colonoscopy on subsequent follow-up. Regular assessment by UGI endoscopy, colonoscopy and USG abdomen is done on each follow-up.

## 3 Discussion

PJS is an inherited autosomal dominant disorder characterized by gastrointestinal hamartomatous polyposis and mucocutaneous pigmentations in the mouth, face, hands, and feet. This rare disorder was first described by Peutz in 1921 and later by Jeghers in the mid-20th century. Its estimated prevalence ranges from 1 in 8300 to 1 in 280,000 individuals [2,4]. It is seen equally in men and women with no racial predominance and diagnosed mostly during childhood to early adulthood [2]. It is attributed to mutations in the serine/threonine kinase 11-LKB1 (STK11/LKB1) tumor suppressor gene located on chromosome 19p13.3 [5]. Approximately half of the patients with PJS will have intussusception because of the large size and pedunculated nature of polyps. Even after surgery, there have been cases of recurrent intussusception mostly due to multiple polyposis in different segments of bowel that needed re-operations [6].

WHO criteria for the diagnosis of PJS include any one of the following: three or more histologically proven Peutz-Jeghers(PJ) polyps, PJ polyps with a family history of PJS, characteristic prominent mucocutaneous pigmentation with a family history of PJS, or PJ polyps with a characteristic prominent mucocutaneous pigmentation [7]. Mucocutaneous pigmentations are due to melanin deposition, often positioned around the lips, buccal mucosa, hands, feet, and sometimes perianal and genital areas. These characteristic macules are seen in almost all patients during infancy; however, they may fade in adulthood and even disappear altogether on rare occasions. In the small intestine, the lifetime risk of developing polyps is about 90 % [8]. Small intestine (jejunum followed by ileum and duodenum) is the most common site of polyp, accounting for 64–96 %. The colon (27–53 %) and stomach (24–49 %) are also affected by polyposis [9]. Extraintestinal polyps, though rare, have also been reported in the gall bladder, ureter, tracheobronchial tree, and tonsils [8].

Bleeding, anemia, intussusception, and intestinal obstruction are common complications associated with hamartomatous polyps [10]. The usual age of presentation following intussusception-related symptoms is in late childhood to early adulthood. Clinical presentations vary according to the site of intussusception, the timing of the presentation, and the possibility of self-reduction. Some common symptoms are colicky abdominal pain, red currant jelly stool, features of acute bowel obstruction, or occult gastrointestinal bleeding [8]. A cohort study by MGF van Lier et al. concluded that the probability of intussusception was at 44 % by age 10 and 50 % by age 20. 69 % had at least one intussusception with the median age at first incidence being 16. Also, the risk of intussusception rises when the polyp size is 15 mm or larger [11]. PJS is associated with a 15-fold increased relative risk of malignancies compared to the general population [12]. Colonic cancer (57 %), accounts for the majority of neoplasm in patients with PJS, followed by breast (45 %), pancreatic (36 %), stomach (29 %), ovary (21 %), small intestine (13 %), and uterine (10 %) tumors [13]. Some extra-gastrointestinal epithelial malignancies of the lungs, thyroid, and ovaries are also reported. Malignancy risk increases with age, with a lifetime risk of 37–93 % [12].

Conventional barium studies, once commonly used to diagnose the cause of partial bowel obstruction, are nowadays seldom performed and have largely been replaced by non-invasive imaging tools like USG and computed tomography (CT). USG shows target or doughnut sign in intussusception, with almost 100 % diagnostic accuracy in children but only 60 % in adults due to the presence of distended, gas-filled intestinal loops [14]. A CT scan is now the preferred diagnostic tool in adult patients with suspected intussusception, which may detect the site and type of intussusception and lead point and identify complications such as intestinal obstruction and bowel ischemia [14]. In patients with suspected PJS or with a strong family history, capsule endoscopy or magnetic resonance enterography is recommended for screening the gastrointestinal tract [15].

According to the American College of Gastroenterology (ACG) clinical guidelines, patients who are affected by PJS should be closely monitored for malignancies of the colon, stomach, small intestine, pancreas, breast, ovary, uterus, cervix, and testes. Large bowel surveillance by colonoscopy or flexible sigmoidoscopy is recommended at 3 years interval from the age of 18. Although there is an increased risk of lung cancer, no particular screening has been suggested. In smokers, it would appear prudent to consider yearly chest radiography or chest CT [15].

Asymptomatic polyps diagnosed during endoscopic screening can be effectively treated with polypectomy, which helps to avoid the need for future urgent operations and the risk of short bowel syndrome with extensive small bowel resections. Small bowels polypectomy can be performed during double-balloon enteroscopy (DBE) or during laparotomy via intraoperative endoscopy. However, in cases where polyps are rapidly growing or associated with intussusception, surgical resection remains the recommended treatment [16]. There is a risk of short bowel syndrome with repeated bowel resection in case of missed polyps or recurrence. Intraoperative endoscopy performed during laparotomy may minimize the need for repeated surgery by visualizing the entire small bowel and removing nearly all the polyps. This “clean sweep” approach is recommended for the management of small intestinal polyps in patients with PJS [17].

## 4 Conclusion

Peutz-Jeghers syndrome presents significant challenges due to its potential for severe gastrointestinal complications and increased cancer risk. Early diagnosis, often aided by characteristic mucocutaneous pigmentation, and ongoing surveillance play key roles in minimizing these risks. The “clean sweep” procedure represents one of the modern approaches to managing this condition. In our case, timely diagnosis and intervention lead to limited segmental jejunal resection preventing more extensive bowel resection and risk of short bowel syndrome.

## Author contribution

1. Constructing hypothesis for the manuscript- Samrat Shrestha, Mecklina Shrestha
2. Planning methodology to reach the conclusion: Samrat Shrestha, Bijay Raj Bhatta, Kaushal Samsher Thapa.
3. Organizing and supervising the course of the article and taking responsibility: Samrat Shrestha
4. Patient follow-up and reporting – Kaushal Samsher Thapa, Mecklina Shrestha, Bijay Raj Bhatta
5. Logical interpretation and presentation of the results- Samrat Shrestha, Kaushal Samsher Thapa, Mecklina Shrestha, Bijay Raj Bhatta
6. Construction of the whole or body of the manuscript- Samrat Shrestha, Bijay Raj Bhatta, Mecklina Shrestha.
7. Reviewing the article before submission not only for spelling and grammar but also for its intellectual content- Samrat Shrestha, Kaushal Samsher Thapa, Mecklina Shrestha, Bijay Raj Bhatta.

## Consent

Written informed consent was obtained from the patient for publication of this case report and accompanying images. A copy of the written consent is available for review by the Editor-in-Chief of this journal on request.

## Ethical approval

The IRB at our institution has waived ethical approval for case reports.

## Guarantor

The guarantor is Samrat Shrestha.

## Funding

There are no sources of funding for this case study to declare.

## Conflict of interest statement

The authors have no conflict of interest to declare.
